# Lateral Entorhinal Cortex is Critical for Novel Object-Context Recognition

**DOI:** 10.1002/hipo.22095

**Published:** 2013-02-06

**Authors:** David IG Wilson, Rosamund F Langston, Magdalene I Schlesiger, Monica Wagner, Sakurako Watanabe, James A Ainge

**Affiliations:** 1 School of Psychology, University of St AndrewsSt Mary's Quad, St Andrews, Fife, United Kingdom; 2 Division of Neuroscience, Medical Research Institute, University of Dundee, Ninewells Hospital & Medical SchoolDundee, United Kingdom

**Keywords:** episodic, memory, hippocampus, associative, context

## Abstract

Episodic memory incorporates information about specific events or occasions including spatial locations and the contextual features of the environment in which the event took place. It has been modeled in rats using spontaneous exploration of novel configurations of objects, their locations, and the contexts in which they are presented. While we have a detailed understanding of how spatial location is processed in the brain relatively little is known about where the nonspatial contextual components of episodic memory are processed. Initial experiments measured *c-fos* expression during an object-context recognition (OCR) task to examine which networks within the brain process contextual features of an event. Increased *c-fos* expression was found in the lateral entorhinal cortex (LEC; a major hippocampal afferent) during OCR relative to control conditions. In a subsequent experiment it was demonstrated that rats with lesions of LEC were unable to recognize object-context associations yet showed normal object recognition and normal context recognition. These data suggest that contextual features of the environment are integrated with object identity in LEC and demonstrate that recognition of such object-context associations requires the LEC. This is consistent with the suggestion that contextual features of an event are processed in LEC and that this information is combined with spatial information from medial entorhinal cortex to form episodic memory in the hippocampus. © 2013 Wiley Periodicals, Inc.

## INTRODUCTION

Episodic memories consist of information about personal events that are rich in contextual detail. To study episodic memory in animals it has been operationalized as memory for spatial locations, stimuli (e.g., objects) encountered within them and the occasion (contextual or temporal) in which the event took place (Clayton and Dickinson, 1998; Eacott and Norman, 2004; Babb and Crystal, 2006). The hippocampus has been implicated in processing episodic memory in humans (Vargha-Khadem et al., 1997; Eldridge et al., 2000; Gelbard-Sagiv et al., 2008) and episodic-like memory in animals (Day et al., 2003; Eacott and Norman, 2004; Fortin et al., 2004; Langston and Wood, 2010). The spatial component of episodic memory, and how it is processed by the brain, has been relatively well characterized in recent years. The discovery of place cells (O'Keefe and Dostrovsky, 1971) and grid cells (Hafting et al., 2005) in the rodent medial temporal lobe has demonstrated that the hippocampus and medial entorhinal cortex (MEC) are key components of a network mediating spatial representations in the mammalian brain. This medial temporal lobe network has been proposed as the neural instantiation of the cognitive map as first described by Tolman (1948) and later developed by O'Keefe and Nadel (1978).

However, memories for specific episodes include contextual information about the occasion in which they took place as well as spatial information. Indeed, numerous studies have shown that rodent hippocampal place cells are influenced by contextual features of an event including nonspatial physical characteristics of an environment (e.g., color) (Anderson and Jeffery, 2003; Leutgeb et al., 2005), cognitive demands (Wood et al., 2000; Ferbinteanu and Shapiro, 2003; Ainge et al., 2007; Griffin et al., 2007; Ji and Wilson, 2008; Ainge et al., 2012; Griffin et al., 2012), internal state (Kennedy and Shapiro, 2009) and olfactory information (Wood et al., 1999).

It has been theorized that the two principal streams of input to the hippocampus via MEC and lateral entorhinal cortex (LEC) provide place cells with spatial and nonspatial (contextual) information respectively, which are integrated into a spatially selective, context-specific response (Hargreaves et al., 2005; Knierim et al., 2006; Hayman and Jeffery, 2008; Hasselmo, 2009; Eichenbaum et al., 2012). Single unit recording studies have demonstrated that LEC neurons lack spatial selectivity (Hargreaves et al., 2005), even in cue rich environments (Yoganarasimha et al., 2011), and instead show preferential activation to objects and their associated places (Deshmukh and Knierim, 2011). However, there has been no attempt to manipulate how stimuli are incorporated with contextual components of environments to assess whether LEC is involved in processing this type of information.

To address this we first examined *c-fos* expression, shown to be critical for learning and memory (Kubik et al., 2007), in the hippocampus and parahippocampal cortices during an object-context recognition (OCR) task. Having found strong c-*fos* expression in LEC during the OCR task, we subsequently carried out a second experiment to determine whether LEC was required for rats to integrate information about objects and the contexts in which they were encountered.

## METHODS

### Experiment 1

#### Subjects

Twenty-one male Lister Hooded rats (Harlan Olac Ltd, Bicester, UK; average weight at start of experiment: 342 g) were subjects in this experiment. They were housed in groups of 3, and kept on a 12 h light/dark cycle. Behavioral testing was carried out during the light phase. Testing was carried out 5 days/week. Compliance was ensured with national (Animals [Scientific Procedures] Act, 1986) and international (European Communities Council Directive of 24 November 1986 [86/609/EEC]) legislation governing the maintenance of laboratory animals and their use in scientific experiments.

#### Apparatus

Behavioral testing took place in a 67 cm square box with 40 cm high walls that could be configured with two sets of contextual features. The first consisted of plain wooden walls and floor painted white. The second context had wall inserts that were covered in granite effect plastic and a gray plastic mesh floor that overlaid the white wooden floor. The box was in a circular curtained arena with prominent extra-maze cues placed on the curtains. These cues were consistently present irrespective of the contextual configuration of the box. Behavior was monitored by an overhead camera. Objects used were 3D household objects that were approximately the same size as a rat in at least one dimension, easily cleanable and made from plastic, metal, glass, or ceramic. They were fixed to the floor of the arena using Dual Lock (3M™, St. Paul, MN).

#### Behavioral testing

**FIGURE 1:**
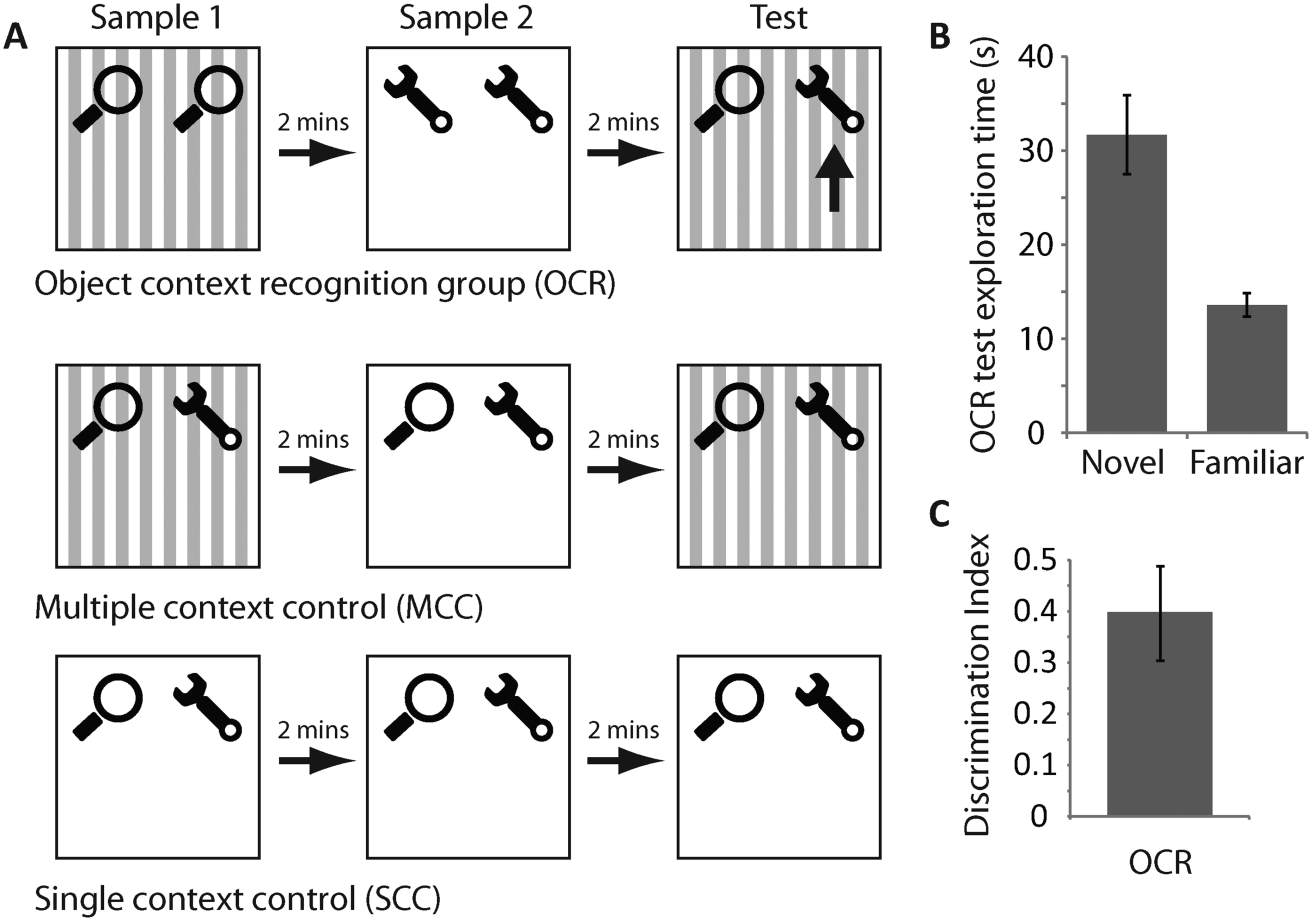
Behavioral measures of novel object-context recognition (Experiment 1). (A) Top row: Object-context recognition (OCR) task. Middle row: multiple context control (MCC). Bottom row: single context control (SCC). (B) Rats in the OCR group spent longer exploring the novel object-context combination than the familiar object-context combination. (C) Data also presented as a discrimination index which was significantly different from chance (*P* < 0.001).

Following 1 week of extensive handling to habituate the rats to the experimenter behavioral testing proceeded in three stages:

1. *Habituation*. Rats were initially habituated for 8 days. On days 1–2 rats were placed in the box for 10 min in their cage groups and allowed to explore. Each cage group experienced each set of contextual features once. On days 3–4 rats were placed in the box for 10 min by themselves and allowed to explore. At the end of the exploration rats were placed in a holding cage for 10 min. Each rat experienced each context once. On days 5–8 rats were placed in the box with 2 novel junk objects and allowed to explore. Each rat experienced each context twice. The junk objects were different every day. Again rats were placed in a holding cage for 10 min after exploration.
2. *Novel object recognition task*. Novel object recognition testing was carried out for 4 days as has been described previously (Ainge et al., 2006). Each rat received two days of testing in each context. Rats were given a 3 min sample trial where they were exposed to two copies of a novel object in one of the contexts and allowed to explore them freely. Sample trials were terminated when rats had accumulated 15 s of exploration time at each object or 3 min, whichever was shorter. Rats were then removed from the box and placed in a holding cage while the box was cleaned and configured for the test trial. Inter-trial interval was ∼1 min. For the test trial a new copy of the object that was presented in the sample trial and a novel object were presented in the same context for 3 min. Exploration of the objects was monitored via an overhead camera linked to a monitor, recorded, and used for analysis. Novel object, side of presentation, and context were counterbalanced within and across days where relevant.
3. *Context manipulations*. Rats were randomly assigned to each group:
  - Novel OCR group (*n* = 8). OCR testing was based on one of the tasks described by Dix and Aggleton (1999). Rats received two sample trials (Fig. 1A; top row); in the first they were presented with 2 identical copies of a novel object in a familiar context. In the second they were presented with 2 copies of a different novel object in a second familiar context. Critically these contexts were in the same physical location and the distal cues in the room were the same for each context allowing the rat to know it was in the same place. Sample trials were terminated when rats had accumulated 15 s of exploration time at each object or 3 min, whichever was shorter. In a test phase the rats were placed in one of the contexts with a copy of each of the objects from the sample trials and exploration of the objects was recorded for 3 min. This tests the rat's memory for object-context associations as one of the objects will have been experienced in this context before while the other will not (see arrow Fig. 1A for novel object-context combination). Test object, test context, order of context presentation in the sample phases and side of presentation were counterbalanced across rats.
  - Multiple context control (MCC; *n* = 7). Rats received two sample trials (Fig. 1A; middle row); in the first they were presented with two different novel objects in a familiar context. In the second they were presented with two different copies of the same objects in the same positions in a second familiar context. Sample trials were terminated when rats had accumulated 15 s of exploration time at each object or 3 min, whichever was shorter. In a test phase the rats were placed in one of the contexts with two different copies of the same objects in the same positions in one of the contexts from the sample trials. In contrast to the OCR group, rats in the MCC group only see familiar object-context associations in the test phase and there is no discrimination between novel and familiar object-context associations. Exploration of the objects was recorded for 3 min.
  - Single context control (SCC; *n* = 6). This is the same as the multiple context control except that the context did not change between trials (Fig. 1A; bottom row). Again in contrast to the OCR group rats in the SCC group only see familiar object-context associations in the test phase and there is no discrimination between novel and familiar object-context associations.

#### Behavioral analysis

To check the reliability of our observation scores a separate observer rescored a subset of videos blind. Blind observer scores were consistently within 10% of the experimenter. Observation scores were converted into discrimination indices to determine the relative exploration of novel versus familiar objects. This removed any bias that may be produced by rats with longer bouts of exploration having a disproportionate effect when comparing total scores:


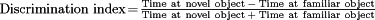


#### Perfusions

One hour following the completion of the behavioral protocol rats were humanely euthanized with i.p. injections of 200 mg/ml/kg sodium pentobarbitone (“Dolethal”, Univet, Bicester, UK). They were then transcardially perfused with 50 ml of 0.9% phosphate buffered saline at a rate of ∼20 ml/min followed by at least 250 ml of 4% paraformaldehyde solution made up in 0.1% phosphate buffer. Brains were then extracted and placed overnight in 20% sucrose solution (made up in 0.1% phosphate buffer).

#### Histology

Brains were cut into 50 μm sagittal sections on a freezing microtome with 1:4 sections being taken for subsequent staining and analysis. To analyse c-*fos* expression the sections were processed immunohistochemically as described previously (Ainge et al., 2004). Sections were washed in phosphate buffer before being placed in blocking solution (20% normal goat serum) for 60 min. Sections were then incubated in anti-*c-fos* primary antibody at a concentration of 1:8000 (Oncogene Research Products, Calbiochem) overnight. Sections were then removed, washed in phosphate buffer and placed in biotinylated IgG (antirabbit, Vectastain Elite ABC kit) at a concentration of 1:200 for 60 min before finally being incubated in avidin–biotin complex (Vectastain Elite ABC kit) at a concentration of 1:50 for a further 60 min. Sections were then reacted with nickel enhanced 3,3-diaminobenzidine tetrahydrochloride (Sigma) before being mounted, dehydrated, and coverslipped with DPX. Sections were analyzed using a light microscope to examine levels of *c-fos* staining as compared to background staining.

To aid anatomical localization of borders between areas a parallel set of sections for each animal was stained with a mouse derived antibody directed against neuronal nuclear protein (NeuN; Chemicon International, Temecula, CA). For the staining protocol see Wilson et al. (2009).

#### Regions of interest

**FIGURE 2:**
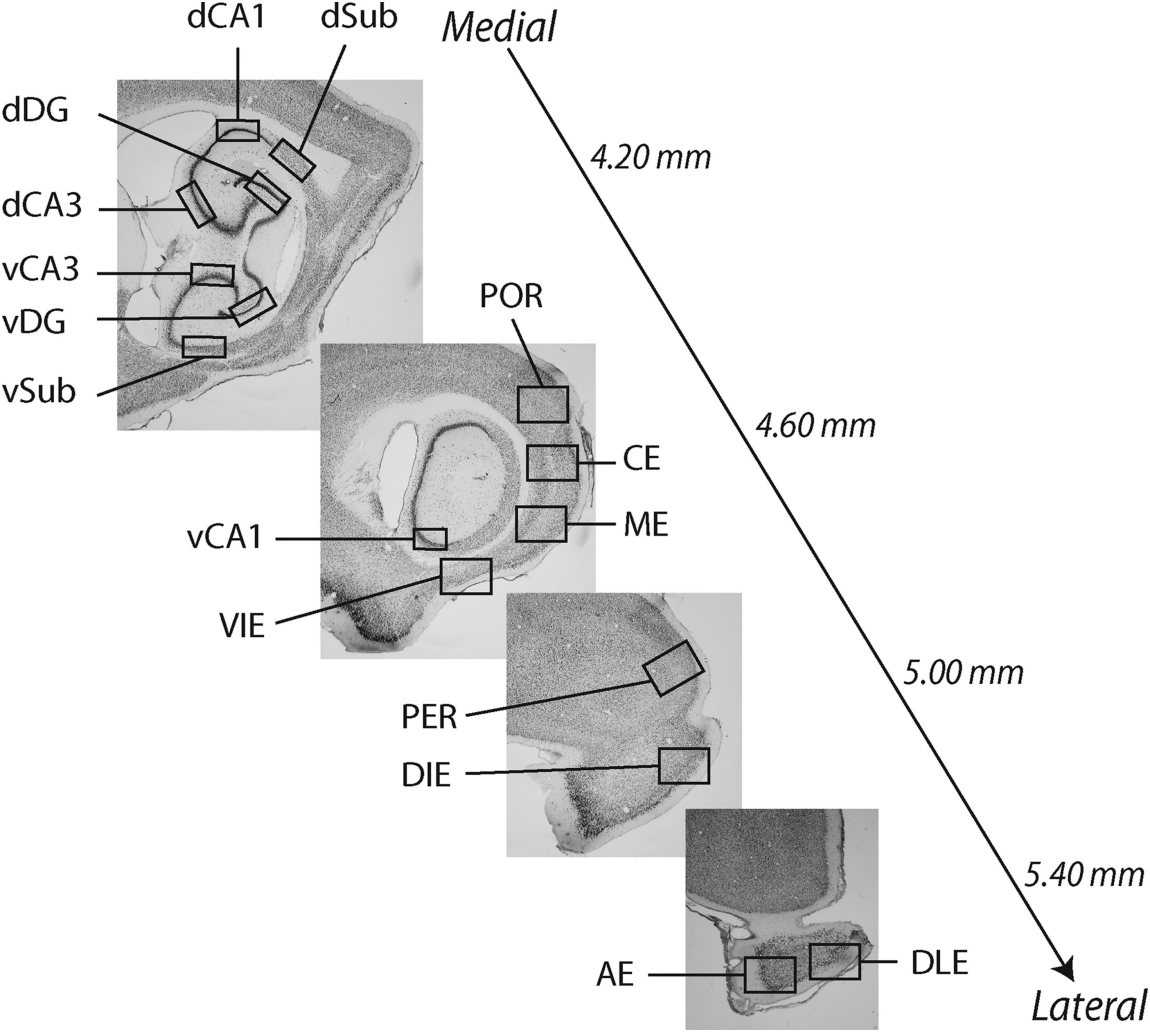
Regions of interest (Experiment 1). Four sagittal sections stained for NeuN in the sagittal plane (figures illustrate the position in the medial-lateral axis relative to midline). Examples of areas from each subregion taken for analysis are labeled. Note that in cortical areas all layers are sampled together while in sections from the hippocampus the sampled areas are confined to the prominent cells layers. Entorhinal cortex subregions; ventral intermediate entorhinal (VIE), dorsal intermediate entorhinal (DIE), dorsal lateral entorhinal (DLE), amygdalo-entorhinal (AE), caudal entorhinal (CE), medial entorhinal (ME). Hippocampus subregions: Dorsal Dentate Gyrus (dDG), CA3 (dCA3), CA1 (dCA1) and Subiculum (dSub) and Ventral Dentate Gyrus (vDG), CA3 (vCA3), CA1 (vCA1), and Subiculum (vSub). Parahippocampal cortices: Perirhinal Cortex (PER), Postrhinal Cortex (POR).

Regions of interest within the *c-fos* labeled sections were identified with reference to the NeuN labeled sections using an on-line atlas of hippocampal anatomy (http://cmbn-approd01.uio.no/zoomgen/hippocampus/home.do (Kjonigsen et al., 2011) combined with Paxinos and Watson (1998). Examples of sampled areas within each subregion are illustrated in Figure 2. Counts were taken from 6 subregions of entorhinal cortex. These included four subdivisions of LEC (ventral-intermediate entorhinal VIE, dorsal-intermediate entorhinal DIE, dorsal-lateral entorhinal DLE, and amygdalo-entorhinal AE) and two subdivisions of MEC (caudal entorhinal CE and medial entorhinal ME). Counts were also taken from two other regions of the parahippocampal cortex (perirhinal and postrhinal) and 8 subregions within the hippocampus (CA1, CA3, DG, and Subiculum in both the dorsal/septal and ventral/temporal hippocampus). As illustrated in Figure 2, all cell layers within the cortical regions were sampled together. This was because the number of *c-fos* positive cells in some layers was very low making comparison between them difficult.

#### c-Fos quantification

*c-Fos* quantification was carried out blind to the experimental condition. Subregions of the hippocampus and parahippocampal cortices were localized using a light microscope at 4× magnification. Photographs of the relevant areas were then taken at 10× magnification with a consistent light level. Photographs of at least four sections were taken for each subregion. For larger subregions up to eight sections were sampled although the number of sections for any given subregion analyzed was constant across rats. Images were processed using Scion Image (v4.0.3.2). *c-Fos* expression was identified by taking a mean gray scale of each image and identifying pixels that were 2 standard deviations darker than the mean. *c-Fos* positive neurons were classified as groups of more than 20 and less than 500 adjacent pixels whose gray scale was more than 2 standard deviations greater than the mean gray scale for that image. To examine density of *c-fos* positive neurons within particular regions the regions of interest were outlined within the section and area of that region measured in mm^2^. Density of *c-fos* expression was then calculated by dividing the total count of *c-fos* positive neurons within each region by the total area from which these counts were taken giving a dependent variable of *c-fos* positive neurons per mm^2^. To allow comparison of regions with different cell densities raw counts from each area were normalized by dividing them by the mean count for that area across groups and multiplying by 100. Statistical analysis was carried out on these normalized scores.

#### Statistical analysis

Normalized *c-fos* positive counts were analyzed in three regional groupings to reduce Type 1 error. These were entorhinal cortex (VIE, DIE, DLE, AE, ME, and CE), parahippocampal cortices (perihinal and postrhinal) and hippocampus (dorsal and ventral portions of CA1, CA3, DG, and subiculum). Counts were analyzed using a repeated measures ANOVA with *Group* (OCR, MCC, and SCC) as the between subjects factor and *Subregion* as the within subjects factor. Following any significant *Group × Subregion* interaction, simple effects were examined using Bonferroni corrected pairwise comparisons to assess how the *c-fos* immunoreactivity within each subregion differed between groups. Significant differences between the OCR group and the controls would demonstrate an effect of discriminating between novel and familiar object-context associations whereas a significant difference between the OCR/MCC and SCC groups would reflect increased contextual processing.

One-sample *t*-tests were performed to determine whether the average discrimination index for the OCR group was different from chance (0). Mean total exploration time in the test phase was analyzed using a univariate ANOVA with *Group* (OCR, MCC, and SCC) as the fixed factor. Mean time to accumulate 15 s exploration at each object in the sample phases was analyzed using a repeated measures ANOVA with *Group* (OCR, MCC, and SCC) as the between subjects factor and *Sample phase* (1 vs. 2) as the within subjects factor. We calculated Spearman's correlation coefficient between the number of *c-fos* immunopositive cells/mm^2^ and the average discrimination index for rats in the OCR group.

### Experiment 2

#### Subjects

Twenty-one male Lister Hooded rats (Harlan Olac Ltd, Bicester, UK; average weight at start of experiment: 408 g) were housed in groups of four under the same conditions as rats in Experiment 1.

#### Surgery

Rats were anesthetized using isoflurane (Abbot Laboratories Ltd, Maidenhead, UK) in an induction box before being placed in a stereotaxic frame (David Kopf, Tujunga, CA) where anesthesia was maintained via a facemask mounted on the incisor bar (2–3% isoflurane, 1.2l/min O_2_). A presurgical analgesic Rimadyl (0.05 ml/rat; 5% w/v carprofen; Pfizer Ltd, Kent, UK) was injected subcutaneously. After shaving the scalp, a midline incision was made and holes were drilled bilaterally at the appropriate stereotaxic co-ordinates (−6.5 mm from Bregma; ±4.5 mm from the midline; −6.4 mm below dura). Dura was cut using the bent tip of a 30 gauge needle and 188 nl of ibotenate (0.03*M* solution in sterile phosphate buffer; Sigma-Aldrich, UK) was infused by pressure ejection from a drawn glass micropipette (tip diameter 30–40 μm) at a 10° (in the ML plane, angled laterally) and left *in situ* for 5 min after infusion. Sham operated controls underwent the identical procedure but received only the vehicle solution (sterile phosphate buffer). Rats were given 7 days to recover from surgery before behavioral testing began.

#### Behavioral apparatus

This was identical to the apparatus described in Experiment 1 except the second context had wall inserts and floor painted with black and white vertical stripes (5 cm width) and black plastic mesh overlaid the floor.

#### Behavioral testing

Following 1 week of extensive handling to habituate the rats to the experimenter behavioral testing proceeded in four stages:

1. *Context habituation*. Rats were initially habituated individually for 5 mins each day over 4 days to the testing box with no objects present and configured to one of two contexts. Rats were placed in a holding cage for 5 min after exploration. Half of the rats experienced the contexts in the order white, stripes, stripes, white, and the other half of the rats experienced stripes, white, white, stripes. *Post hoc* analyses of habituation to the two contexts served as a measure of context recognition.
2. *Habituation to objects in the box*. For the next 4 days rats were individually placed in the box with 2 novel objects (replaced each day) and allowed to explore for 5 min. Each rat experienced each context twice. The junk objects were different every day. Again rats were placed in a holding cage for 5 min after exploration.
3. Novel object recognition task, as described in Experiment 1.
4. *Novel OCR task*. These procedures were the same as those described in Experiment 1 for the novel OCR group (see Fig. 1A) with the exception that the task repeated over 4 days to enable counterbalancing. Within lesion and sham groups we counterbalanced the order of contexts used in sample phases, the test context and the side for the novel object-context association in the test phase.

#### Perfusions

As described in Experiment 1.

#### Histology

We immersed the brains in egg yolk within 24-well tissue culture plates containing paraformaldehyde (40%) in the empty neighboring wells for 5 days to fix the egg onto the outside of the brains. We then cut the brains into 50 μm coronal sections on a freezing microtome and took 1:4 sections for subsequent staining and analysis. Sections were stained with cresyl violet, mounted onto slides and coverslipped using DPX.

#### Lesion data analysis

We viewed slides under a light microscope (Leitz Diaplan) and judged lesion extent by the lack of cell bodies or by cells that were shrunken and damaged. We drew lesion damage onto ten standardized sections of LEC (ranging from −7.66 to −4.42 mm from Bregma, using Scion Image (v4.0.3.2) and calculated the total area of damage in pixels across both hemispheres for each of the subregions of LEC (VIE, DIE, DLE, and AE) and the subregions combined (the whole LEC). We then converted this area into a percentage of the total LEC pixel area across both hemispheres.

#### Behavioral data analysis

To check for reliability a separate observer rescored a subset of videos “blind” for each task and these scores were found to be consistently within 10% of the experimenter's. For the context recognition task, videos were observed offline and viewed through a 3 × 3 box grid acetate drawing placed on the monitor screen to overlay the behavioral testing box. When the rat's front and hind legs had moved through a grid box we added one to the exploration count. For all other tasks we converted observation scores into discrimination indices as in Experiment 1.

#### Statistical analysis

For the novel object recognition and novel OCR tasks separate univariate ANOVAs were used to compare the average discrimination indices and total exploration times (time at novel + time at familiar objects) in the test phase between lesion and sham groups. For the novel OCR task the mean time to accumulate 15 s exploration at each object in the sample phases was analyzed using a repeated measures ANOVA with *Group* (lesion vs. sham) as the between subjects factor and *Sample trial* (1 vs. 2) as the within subjects factor. For the novel object recognition task the mean time to accumulate 15 s exploration at each object in the sample phase was analyzed using a univariate ANOVA with *Group* as the fixed factor. One-sample *t*-tests were used to determine whether the average discrimination index over the four days for each group was different from chance (0). We calculated Spearman's correlation coefficient between the extents of the lesion damage (% damage; lesioned rats only) to the whole LEC or to each of its subdivisions (VIE, DIE, AE, and DLE) and the average discrimination index for the four days of the object-context task and object recognition task.

To assess the ability of rats to remember different contexts a repeated measures ANOVA was carried out on average exploration scores across the four days of habituation with a between-subjects factor of *Group* (lesion or sham). Differences in habituation across the four days could be driven by two processes. The first is habituation to general testing procedures (e.g., handling, transportation from the holding room etc.) and the second is habituation to the specific testing contexts. Habituation to general testing procedures should take place gradually across the four days. To test this paired *t*-tests were then used to compare the effects of habituation to the task (day 1–day 2; day 3–day 4). Habituation to the specific contexts, however, would take place between the first and second exposure to the context. The order of presentation of the contexts was white, stripes, stripes, white (or vice versa). Consequently, to test for habituation to context a second set of paired *t*-tests were used (day 1–day 4; day 2–day 3). We also assessed whether different contexts could influence exploration to provide another measure of context recognition. To do so, we conducted a repeated measures ANOVA on average exploration scores with Context (white vs. stripes) and Familiarity (novel vs. familiar) as within-subject factors with *Group* (lesion vs. sham) as a between-subjects factor. We additionally applied the same test separately for lesioned and sham-lesioned rats to determine whether lesioned rats alone were influenced by context. The Huyn-Feldt correction was applied to all ANOVAs.

## RESULTS

### Experiment 1

Rats in the novel OCR group (Fig. 1A; top row) explored the novel configurations of objects and contexts in preference to previously experienced configurations and thus demonstrated memory for object-context associations (*t*_(7)_ = 8.714, *P* < 0.001; Figs. 1B,C). We compared *c-fos* expression in rats from this group with two control groups to compare how the network responded to discrimination of novel versus familiar object-context associations (OCR) as compared to exposure to object-context configurations that did not allow for discrimination between novel and familiar object-context associations (Fig. 1A; middle and bottom rows). In the multiple context control group (MCC) rats experienced the same objects and contexts as the novel OCR group but the objects were consistently in the same place irrespective of context. In the SCC group rats experienced the same objects in the same positions as the MCC group but only experienced one context. Consequently, unlike the novel OCR group, the MCC and SCC groups never had the opportunity to discriminate novel versus familiar combinations of object and context.

**FIGURE 3:**
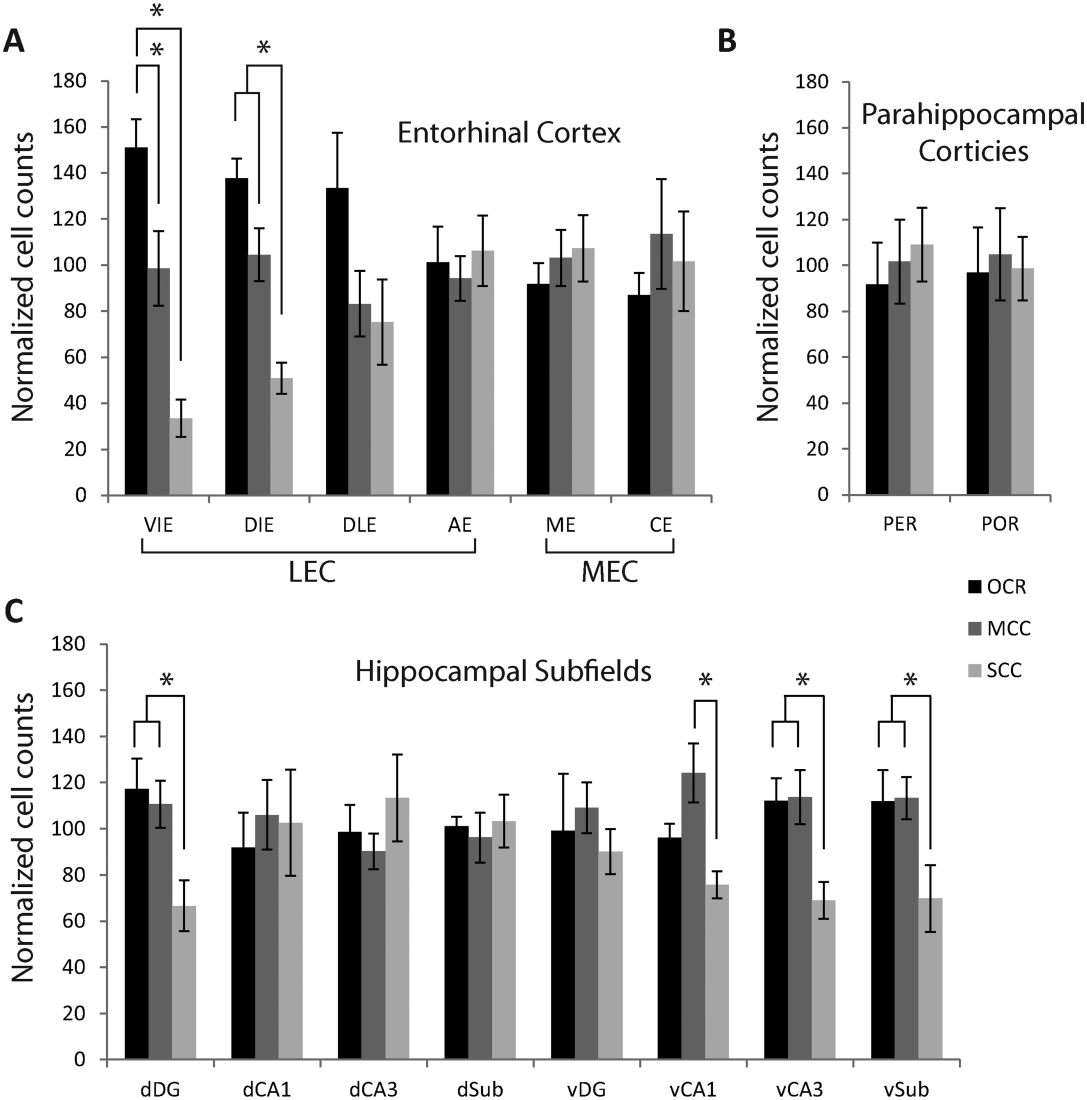
Network activation during novel object-context recognition (OCR), multiple context control (MCC) and single context control (SCC) conditions (Experiment 1). (A) Normalized *c-fos* expression in the entorhinal cortex subdivided into subregions comprising the LEC (VIE, DIE, DLE, and AE) and MEC (ME and CE). (B) Normalized *c-fos* expression in the peri- and postrhinal cortices. (C) Normalized *c-fos* expression in the hippocampus. Asterisks refer to statistically significant (*P* < 0.05) Bonferroni-corrected pairwise comparisons.

**FIGURE 4:**
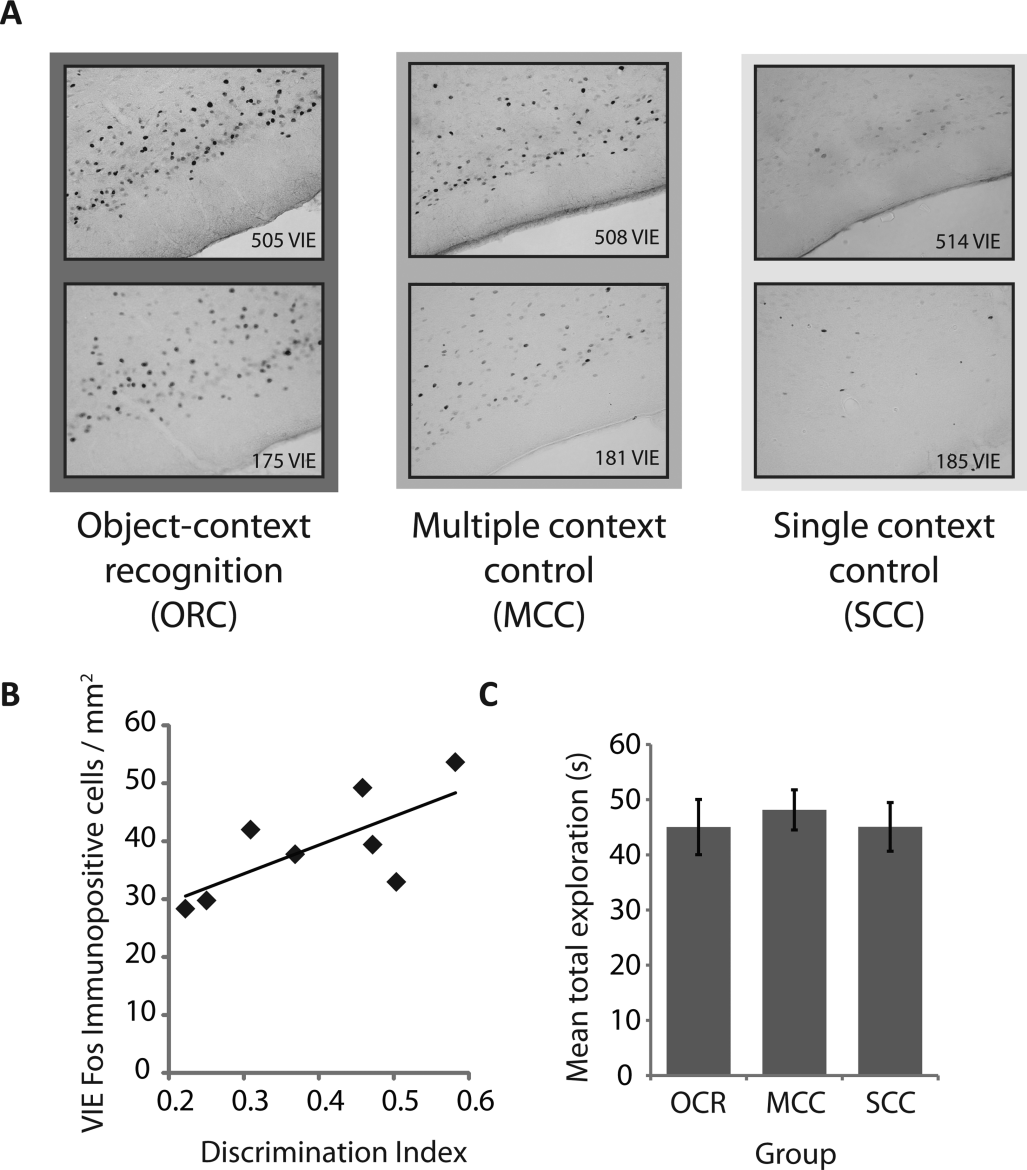
LEC activation during novel object-context recognition (Experiment 1). (A) *c-Fos* expression in VIE portion of LEC was significantly greater in the novel object-context recognition group (OCR) relative to the multiple context control (MCC) and the single context control (SCC). A further significant increase in *c-fos* expression in VIE was found in the MCC versus the SCC. Photographs show examples of *c-fos* expression from VIE of animals in the different conditions. (B) Discrimination of the novel versus familiar object-context association was correlated with level of *c-fos* expression in VIE in the OCR group (*P* = 0.049). (C) Mean total exploration of objects in the test phase did not differ across the three groups.

*c-Fos* expression was quantified throughout the hippocampus, entorhinal, perirhinal, and postrhinal cortices (Fig. 3). Figure 3 illustrates that there was differential *c-fos* immunoreactivity across groups within subregions of the entorhinal cortex and hippocampus. This was confirmed by repeated measures ANOVA of the entorhinal data that revealed a significant effect of *Group* (F_(2,18)_ = 4.67, *P* = 0.026, partial η^2^ = 0.38) and a significant *Group × Subregion* interaction (F_(10,90)_ = 3.54, *P* < 0.001, partial η^2^ = 0.32). Bonferroni corrected pairwise comparisons revealed that *c-fos* expression in the VIE subregion of LEC was significantly greater in the OCR group relative to both controls (Fig. 4A). This suggests a role for the LEC, and the VIE in particular, in recognizing object-context associations. Moreover, there was a statistically significant positive correlation between stronger discrimination of novel versus familiar object-context associations and *c-fos* expression in VIE (*r* = 0.71, *P* = 0.049; Fig. 4B). The OCR and MCC groups had significantly greater *c-fos* immunoreactivity (*P* < 0.05) than the SCC group in the DIE subregion of LEC demonstrating that the DIE has greater *c-fos* expression in conditions where multiple sets of contextual features were experienced. Critically, the total amount of exploration was not significantly different across the groups in either the sample (*F*_(2,18)_ = 0.42, *P* = 0.66) or test phases (F_(2,18)_ = 0.89, *P* = 0.92; Fig. 4C), which rules out the possibility that differential *c-fos* expression was due to different levels of interaction with the objects. No significant differences were seen in the DLE or AE suggesting that subregions of LEC that project to ventral rather than the dorsal hippocampus are involved in processing contextual information. No significant differences were seen between groups in MEC.

To examine how the rest of the parahippocampal cortices process contextual information we also measured *c-fos* expression in the perirhinal and postrhinal cortices and found no significant differences in these areas across the three behavioral conditions (Fig. 3). We went on to examine *c-fos* expression in the different subfields of the hippocampus. Repeated measures ANOVA revealed a significant *Group × Subregion* interaction (*F*_(14,126)_ = 1.96, *P* = 0.031, partial η^2^ = 0.22). Bonferroni corrected pairwise comparisons revealed that *c-fos* expression in the ventral portions of CA3, CA1 subiculum and in dorsal dentate gyrus showed significantly (*P* < 0.05) more activation in conditions with multiple contexts (OCR and MCC) relative to a single context (SCC; *P* < 0.05; Fig. 3). *C-Fos* expression in the dorsal portions of CA3, CA1, and subiculum and in the ventral DG did not differ across conditions.

### Experiment 2

#### Histology

**FIGURE 5:**
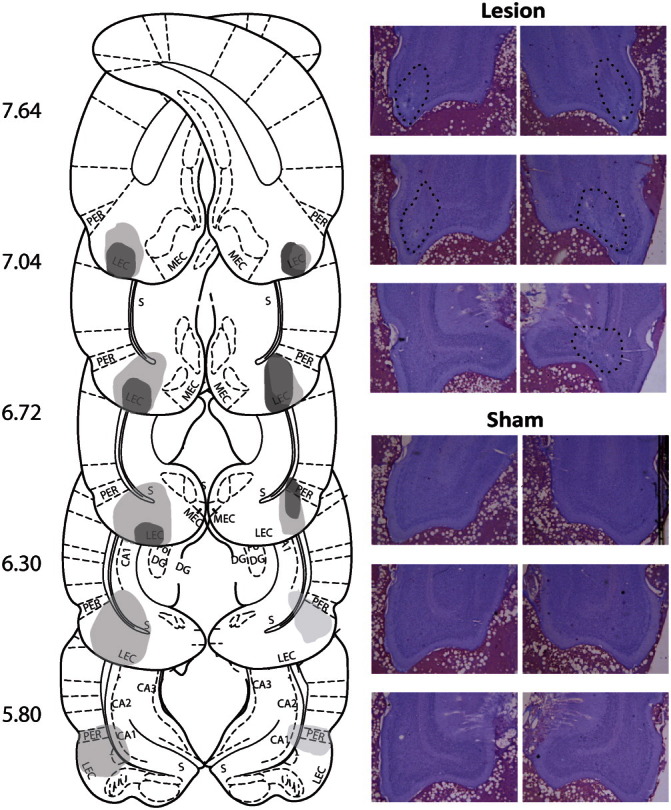
Examples of lesion damage extent in Experiment 2. (Left) Schematic representation of lesion damage from rats with the largest (light gray; rat 2 in Table 1) and smallest (dark gray; rat 4 in Table 1) lesion to LEC. Representations of coronal sections adapted from Paxinos and Watson (1998). Numbers on the left represent the distance from Bregma (mm) (Right) Photograph example of a bilateral LEC lesion (top three pairs of photographs; rat 3 in Table 1) compared to a sham LEC lesion (bottom three pairs of photographs). Photographs were taken through a light microscope using a ×2.5 objective. Dashed black lines surround areas of lesion damage. [Color figure can be viewed in the online issue, which is available at wileyonlinelibrary.com.]

Thirteen of the fourteen rats in the lesion group had lesion damage to the LEC, of which 5 were classified as unilateral lesions. The average bilateral damage per rat (including those with unilateral lesions) was 33% (±4 SEM) within which the relatively greatest damage was DIE > VIE > AE > DLE (Table 1 and Fig. 5). In most rats there was some minor damage to ventral subiculum, CA1, MEC, and/or perirhinal cortex although this was estimated to be <5% damage of their total area (e.g., see external damage present in the largest lesion depicted in Fig. 5). From the 7 rats with sham lesions there was no damage to LEC or surrounding regions.

**TABLE 1:** Extent of Bilateral Lesion Damage to the LEC and its Subregions

Rat	Classification	LEC (%)	VIE (%)	DIE (%)	DLE (%)	AE (%)
1	Unilateral	21.26	36.24	27.48	8.80	0.00
2	Bilateral	60.53	49.47	83.05	54.48	50.00
3	Bilateral	39.84	66.03	62.93	10.72	6.64
4	Bilateral	13.08	15.48	24.76	5.05	0.00
5	Bilateral	43.80	59.61	57.80	25.42	50.00
6	Unilateral	32.55	33.16	49.20	21.83	50.00
7	Bilateral	48.42	33.50	70.47	44.89	43.64
8	Unilateral	18.09	6.86	31.75	17.23	12.51
9	Bilateral	35.65	18.13	37.44	46.63	0.00
10	Bilateral	40.92	21.15	54.31	44.22	82.36
11	Unilateral	14.46	14.95	21.54	10.39	0.00
12	Bilateral	44.67	20.95	71.33	45.10	0.00
13	Unilateral	17.04	21.02	32.48	4.46	50.00
**Average**		**33.10**	**30.50**	**48.04**	**26.09**	**26.55**

Each number reflects the percentage area of damage relative to the intact area of a sham lesioned rat for each rat classified with a unilateral or bilateral lesion. Average percentages for the LEC lesion group are shown in bold in the bottom row.

#### Impaired object-context recognition

**FIGURE 6:**
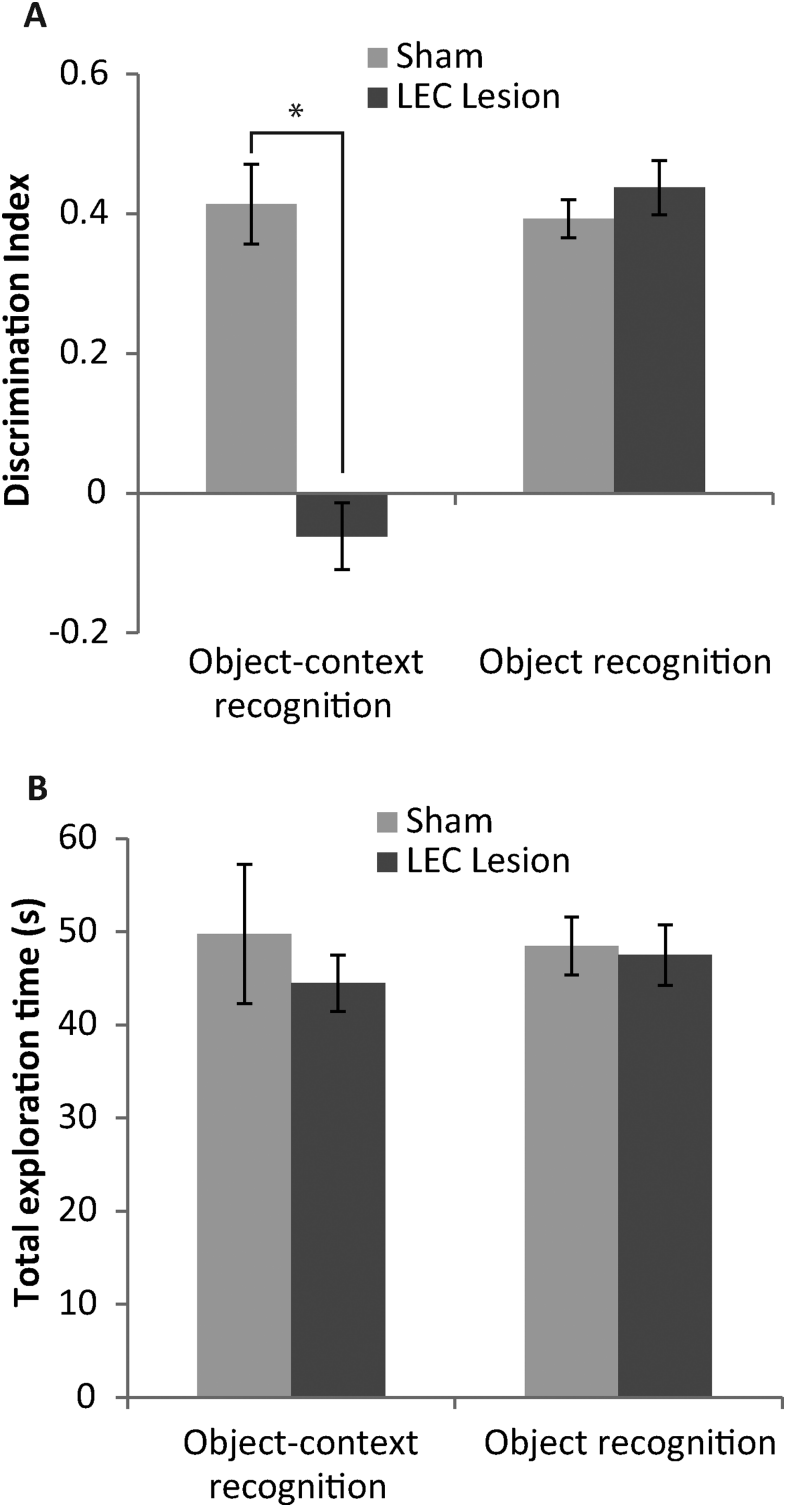
Performance of rats with sham and LEC lesions during the novel object-context recognition and novel object recognition tasks (Experiment 2). (A) In the novel object-context recognition task sham-operated control rats showed a clear preference for exploring the novel combination of object and context whereas rats with LEC damage explored both combinations equally. In the novel object recognition task both groups preferred to explore novel objects relative to familiar objects. (B) Rats in sham and LEC lesion groups showed no difference in the total amount of time exploring objects (time spent at novel + familiar objects) in either task.

Discrimination indices were significantly different between sham and LEC lesion groups in the novel OCR task (*F*_(1,18)_ = 37.796, *P* < 0.001, partial η^2^ = 0.677; Fig. 6A). Rats in the sham group had discrimination indices significantly greater than chance (*t*_(6)_ = 7.255, *P* < 0.001) demonstrating memory for the familiar object-context associations. Rats in the LEC lesion group, however, showed no preference for novel versus familiar object-context associations (*t*_(12)_ = −1.297, *P* = 0.219) demonstrating a critical role for the LEC in processing objects in context. The total time spent exploring objects was comparable between rats with LEC lesions and rats with sham lesions in both the test phase (*F*_(1,18)_ = 0.597, *P* = 0.45; Fig. 6B) and the sample phases (*F*_(1,18)_ = 0.191, *P* = 0.67) demonstrating that rats with LEC lesions were as interested in exploring objects in general as sham-lesioned rats.

#### Normal object recognition

One interpretation of the data from the rats with LEC lesions is that they do not discriminate between novel and familiar object-context combinations because they cannot discriminate between objects. Another is that the LEC lesion destroyed their natural propensity for novelty seeking behavior. To address these potential caveats we examined the ability of rats with LEC lesions, relative to control rats, to discriminate between novel and familiar objects in a standard test of novel object recognition. ANOVA revealed that discrimination indices for novel object recognition were not different between sham and LEC lesion groups (*F*_(1,18)_ = 0.466, *P* = 0.504; Fig. 6A). Discrimination indices were significantly different from chance showing that both groups showed preferential exploration of the novel object (Sham: *t*_(6)_ = 14.40, *P* < 0.001; LEC Lesion: *t*_(12)_ = 10.46, *P* < 0.001) and thus remembered familiar objects. Again, there was no difference between rats with LEC lesions and rats with shams lesions in their general exploration of objects since they spent similar total amounts of time exploring objects in the test phase (*F*_(1,18)_ = 0.037, *P* = 0.850; Fig. 6B) and the sample phase (*F*_(1,18)_ = 0.02, *P* = 0.96). These data demonstrate that the inability of rats with LEC lesions to remember object-context associations is not simply due to an inability to remember object identity or a lack of novelty-seeking behavior.

#### Normal context habituation

**FIGURE 7:**
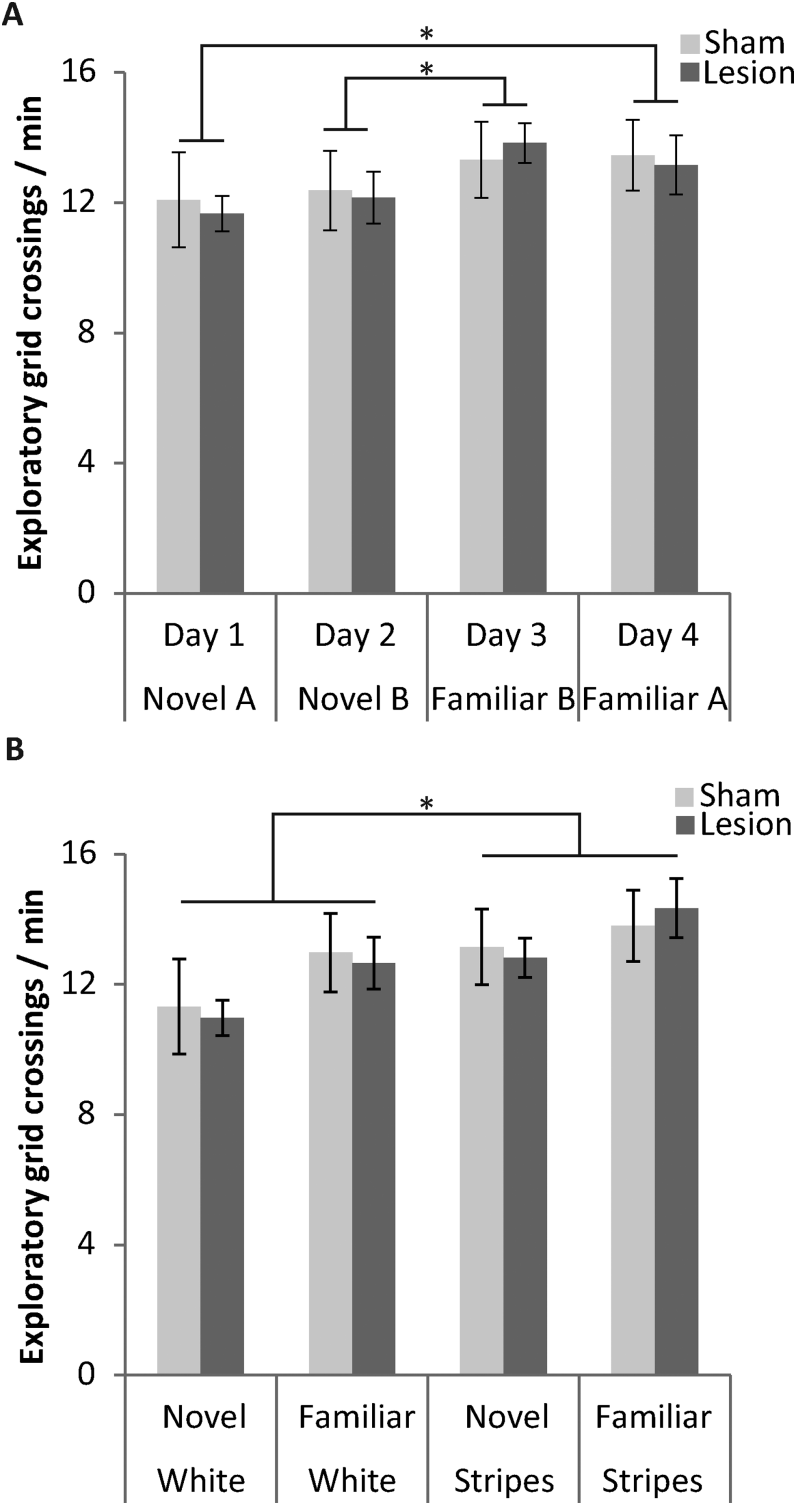
A. Exploration rates across the first four days of habituation to contexts (no objects present) for rats with sham and LEC lesions (Experiment 2). On day 1 rats experienced the novel Context A, which was either white or stripes, counterbalanced across rats; on day 2 rats experienced the other novel context (Context B); on day 3 rats received the Context B again; on day 4 rats received Context A again. Note the significant difference between day 1 versus 4 and day 2 versus 3. Combined with the lack of difference between days 1 versus 2 and days 3 versus 4 this shows habituation to context. (B) Exploration rates across the different contexts. Note that rats in both groups explore more in familiar than novel contexts and that both groups show different amounts of exploration in the different contexts. This shows that the rats differentiate between contexts and remember a previously experienced context.

Another interpretation of the data is that LEC lesioned rats were simply unable to remember or discriminate between novel and familiar contexts. To address this we assessed rats' exploratory behavior during habituation to the contexts. Rats explored at different rates across the first four days of habituation (*F*_(3,50)_ = 3.187, *P* = 0.035, partial η^2^ = 0.150; Fig. 7A). These differences were not suggestive of an overall order effect caused by habituation to the general experimental procedures since rats explored similarly between days 1 and 2 (*t*_(19)_ = −0.680, *P* = 0.505) and between days 3 and 4 (*t*_(19)_ = 0.720, *P* =0.480; Fig. 7A). Instead, the differences were indicative of habituation to specific contexts since rats explored more during their second exposure to a context as compared to their first: exploration was greater in context 1 on day 4 compared to context 1 on day 1 (*t*_(19)_ = −2.329, *P* = 0.031) and in context 2 on day 3 as compared to context 2 on day 2 (*t*_(19)_ = −3.412, *P* = 0.003); Fig. 7A). Importantly, there was no main effect of Group (*F*_(1,18)_ = 0.009, *P* = 0.927; Fig. 7A) or Group × Day interaction (*F*_(3,50)_ = 0.216, *P* = 0.871) meaning that rats in lesion and sham groups explored the contexts similarly and that the ability to recognize a previously experienced context was unimpaired in rats with LEC lesions.

We also assessed whether different contexts had any differential effect on exploration during the habituation phase. We found that across the four days of habituation there was a main effect of Context (white vs. stripes [*F*_(1,18)_ = 15.903, *P* = 0.001, partial η^2^ = 0.469], a main effect of Order (novel vs. familiar [*F*_(1,18)_ = 7.895, *P* = 0.012, partial η^2^ = 0.305]) but no significant effects of Group (lesion vs. sham [*F*_(1,18)_ = 0.010, *P* = 0.922), nor any Group interactions. These analyses demonstrate that both groups show differential exploration of the contexts and that their levels of exploration change during their second experience of a context. To further examine the abilities of the two groups to recognize a previously experienced context separate ANOVAs on the lesion and sham group were carried out. ANOVA of the lesion group found a significant effect of Context (*F*_(1,12)_ = 12.310, *P* = 0.004, partial η^2^ = 0.506) and Order (*F*_(1,12)_ = 6.058, *P* = 0.030, partial η^2^ = 0.335) and a separate ANOVA on the sham group found a significant effect of Context (*F*_(1,6)_ = 7.600, *P* = 0.033, partial η^2^ = 0.559) and a trend towards an effect of Order (*F*_(1,6)_ = 4.347, *P* = 0.082, partial η^2^ = 0.420). Together, these data demonstrate that rats in both lesion and sham groups modified their exploration depending upon context familiarity as well as by the type of context. This demonstrates that rats with LEC lesions could recognize different contexts.

#### Variability in lesion size did not correlate with behavior

**FIGURE 8:**
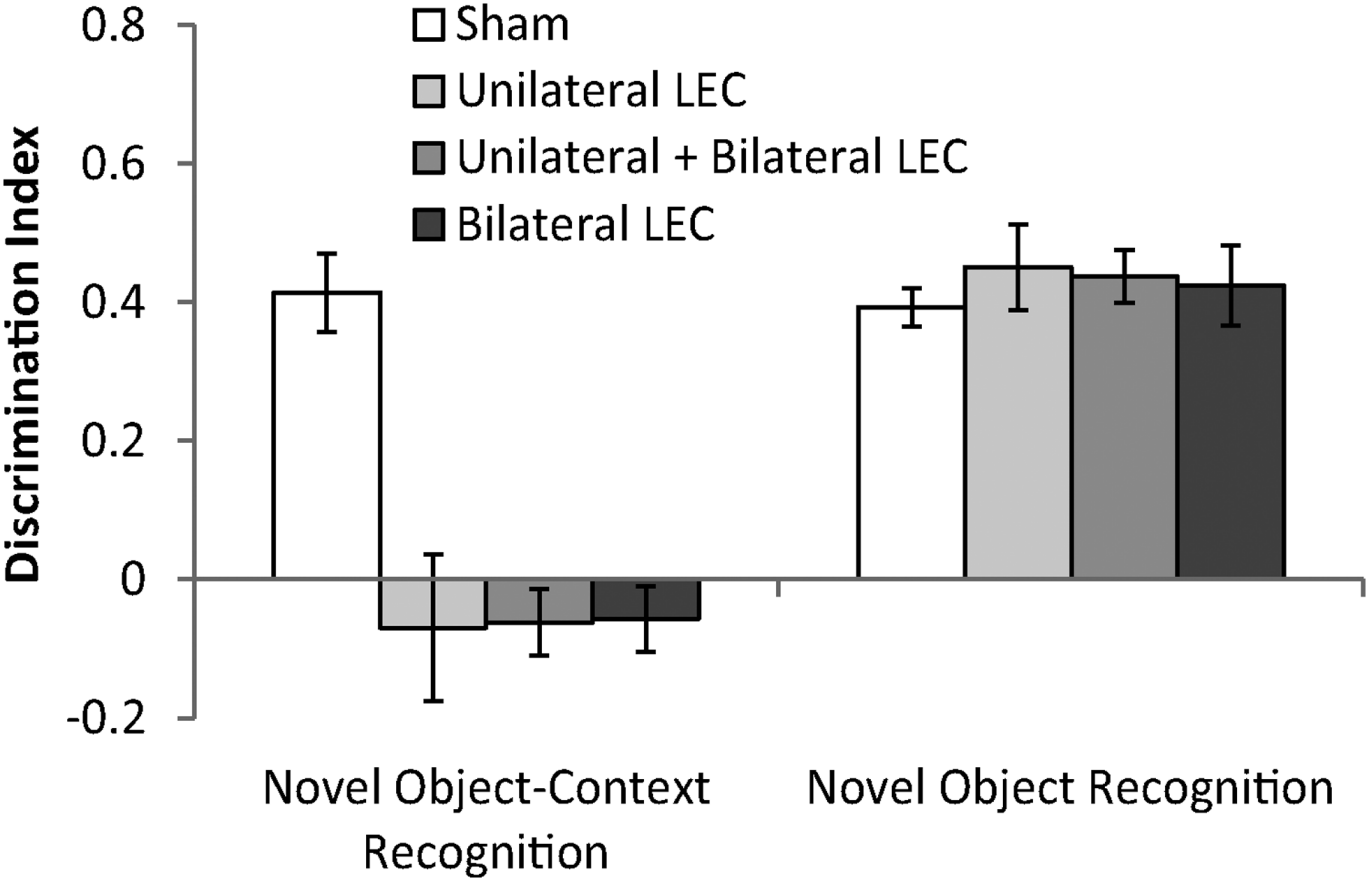
Comparison of unilateral (*n* = 5) and bilateral (*n*= 8) lesioned rats with sham (*n* = 7) and the combination (unilateral + bilateral) lesioned group (*n* = 13) used in the main analyses (Experiment 2) within the novel object-context recognition and novel object recognition tasks.

There was some variability in the size of the LEC lesions (Table 1) with some rats having extensive bilateral damage (*n* = 8) and some only unilateral damage (*n* = 5). Clearly, this could affect the memory ability of the rats. We tested this by examining the memory performance of rats with bilateral relative to unilateral lesions and by correlating the memory performance of the rats with total lesion damage. Discrimination indices were not statistically different between unilateral and bilateral LEC lesion groups during novel object recognition (*F*_(1,11)_ = 0.090, *P* = 0.770) or novel OCR (*F*_(1,11)_ = 0.017, *P* = 0.900; Fig. 8), nor were there any significant correlations between discrimination indices (for novel object recognition or novel OCR) and the extent of lesion damage to the whole LEC or any of its subdivisions (VIE, DIE, DLE, or AE).

## DISCUSSION

These experiments sought to examine the role of the LEC in processing nonspatial contextual features of an environment during recognition memory. Consistent with previous work (Dix and Aggleton, 1999; Mumby et al., 2002a; Eacott and Norman, 2004) it was shown that rats will explore novel object-context combinations in preference to familiar combinations, demonstrating memory for previously encountered object-context associations. Rats that discriminated novel versus familiar object-context associations had greater *c-fos* expression in the LEC than rats who were presented with objects and contexts that were consistently paired. This level of discrimination was significantly correlated with *c-fos* expression in LEC such that greater activation was associated with stronger discrimination. No other regions sampled showed significantly increased activation when discriminating novel versus familiar object-context associations. Additionally, *c-fos* expression in LEC and subregions of the hippocampus was increased following exposure to multiple versus single sets of contextual cues. A subsequent lesion experiment demonstrated that the LEC was critical for novel OCR but not required for independent object or context recognition.

Together, the effects of greater *c-fos* activation in LEC during OCR and of impaired OCR in LEC lesioned rats demonstrate that the LEC is required for OCR. These effects were not a consequence of impaired independent recognition of objects or contexts or of any altered motivation to explore novelty. The pattern of increasing *c-fos* activation in LEC across the three conditions of Experiment 1 (OCR > MCC > SCC) may provide an additional insight into the role of the LEC in processing contextual information. If one considers objects and contexts as part of an overall contextual environment our findings may reflect a role for the LEC in binding objects with the contexts in which they are experienced to form a representation of a new contextualized environment. Thus, in the SCC group (Experiment 1) rats experienced only one new environment that required the binding of objects to their associated context. Similarly, in the context habituation of Experiment 2 only one environment was experienced per session and no objects to process. However, in the MCC group (Experiment 1) two out of three of the environments were new and these required the binding of objects to their associated context. Finally, in the novel OCR test (Experiments 1 and 2) there were three new environments to process per session and again these involved binding objects to context. If the role of LEC is indeed to create representations of new contextualized environments by binding objects to contexts then it would be activated in the manner it was in Experiment 1 (activation in OCR>MCC >SCC) and would need to be intact to facilitate novel OCR, as was the case in Experiment 2.

The importance of the inclusion of objects within contexts to drive LEC activity complements single-neuron studies demonstrating that LEC neural activity is correlated with object processing (Deshmukh and Knierim, 2011; Deshmukh et al., 2012). Since lesions to perirhinal cortex, one of the main afferents of LEC, produce a deficit in object recognition memory (Bussey et al., 1999; Brown and Aggleton, 2001; Murray and Richmond, 2001; Mumby et al., 2002b; Warburton et al., 2003), a possible interpretation of our data is that the LEC provides the link between object identity processed in perirhinal cortex and episodic memory processed in the hippocampus by placing objects within the context in which they were experienced.

Examination of the hippocampus and other parahippocampal areas indicate that dorsal DG and subregions of the ventral hippocampus also showed greater activation in conditions where multiple contexts were experienced (OCR and MCC) relative to conditions in which only one context was experienced (SCC). This is consistent with previous reports showing that ventral hippocampus has a role in processing nonspatial information (Bannerman et al., 2002; Kjelstrup et al., 2002) and is less critical for spatial learning and memory than dorsal hippocampus (de Hoz et al., 2003; Moser et al., 1993; Moser and Moser, 1998). There are a number of studies that have implicated the hippocampus in processing contextual information (Mumby et al., 2002a; Maren, 2008; Rudy, 2009; Sill and Smith, 2012). Our current data would suggest that these effects are most likely mediated through the LEC, DG, and ventral hippocampus.

Although the hippocampus is involved in processing contextual information, it has previously been reported that it is not *necessary* for memory of object-context associations (Eacott and Norman, 2004; Langston and Wood, 2010). We wanted to examine whether the increased LEC activation in rats demonstrating memory for object-context associations, as suggested by increased *c-fos* expression, is a critical mechanism for remembering objects in context. Damage to the LEC produced a profound inability to associate object identity with the contextual features of the environment. This is consistent with the suggestion that the LEC is a critical component of the network responsible for processing nonspatial contextual features of an environment. Importantly, rats with LEC lesions showed normal object recognition and habituation to contexts, demonstrating that this effect was not due to an inability to remember previously experienced objects or contexts individually, or an altered motivation to explore novelty.

Interestingly, rats with unilateral lesions of LEC were equally impaired in novel OCR as those with bilateral lesions. Although this is unusual it is not without precedent. Unilateral amygdala lesions cause a severe deficit in contextually cued fear memory (Flavell and Lee, 2012), inactivating hippocampus bilaterally versus unilaterally produced comparable impact on spatial memory consolidation (Cimadevilla et al., 2008) and unilateral dopamine lesions had bilateral effects on monoamine levels (Pierucci et al., 2009). Moreover, fMRI studies in humans have shown that normal functioning LEC hemispheres are highly functionally connected (Lacy and Stark, 2012). Thus, it could be the case that physical damage to one LEC hemisphere caused a bilateral functional impairment.

Additionally, the extent of lesion damage to the LEC as a whole (or to each of its subdivisions) was not correlated with memory impairments. The intrinsic connectivity of the LEC is beginning to be better understood and it is now clear that there are extensive connections between layers in LEC although these interconnections tend to be within segregated populations that project to different areas of the hippocampus (Canto et al., 2008; Canto and Witter, 2012). For example, the area of LEC (VIE and to a lesser extent DIE) that projects to ventral hippocampus has strong intrinsic connectivity while having much weaker connectivity with the area of LEC that projects to dorsal hippocampus (DLE). This is all consistent with the suggestion that the ventral hippocampus and the VIE form a functional network. The interconnectivity of this network might explain why small lesions of LEC produce functional deficits. Considering both experiments together, the VIE and possibly the DIE, but not the DLE or AE, are activated during OCR and combined lesions of DIE, VIE, AE, and DLE (in that order of relatively greatest damage) produced a clear deficit in OCR. This suggests that the areas of LEC connecting with ventral hippocampus (VIE/DIE) are necessary for OCR and the DLE is not. However, it is not until future studies directly compare OCR in rats with either VIE/DIE or DLE lesions that this can be determined more definitively.

So far we have emphasized the role of the LEC in processing nonspatial information. Our data show that *c-fos* expression in LEC is increased when rats discriminate between combinations of stimuli that cannot be discriminated using spatial information and that rats with lesions of the LEC cannot use this nonspatial information to make these discriminations. However, while it is clear that LEC is necessary for processing nonspatial information it is not yet clear whether it might also be involved in processing spatial information. Indeed some recent data suggest that the LEC might be involved in processing the association of objects and the places in which they were experienced (Deshmukh and Knierim, 2011; Van Cauter et al., 2012). Given that some researchers have suggested that spatial and contextual information may be processed together (Eichenbaum et al., 2012) a further intriguing hypothesis is that the LEC may be involved in associating contextual features with spatial locations as well as its role in object-context associations described in the current study.

Another interesting question is the role of the MEC in processing contextual information. It has been suggested by some authors that the MEC may process contextual information (Eichenbaum et al., 2012) and this is consistent with one report showing a deficit in object-context memory following lesions of the postrhinal cortex which provides input to the MEC (Norman and Eacott, 2005). This account would suggest that increased *c-fos* expression in LEC in the novel OCR group may be due to increased activation from MEC efferents to LEC. However, we found no increase in *c-fos* expressing neurons in MEC. Moreover, this would not account for the object-context memory impairment reported here by lesions of the LEC. Therefore, our data clearly implicate the LEC, rather than MEC, in nonspatial, contextual processing.

The necessary role for LEC in novel OCR presented here furthers our understanding of the pathology of memory-related deficits in Alzheimer's Disease since patients with Alzheimer's Disease (from very mild stage through to late stage) suffer striking degeneration of neurons in entorhinal cortex (Hyman et al., 1986; Braak and Braak, 1991; Gomez-Isla et al., 1996; Price et al., 2001; Stranahan and Mattson, 2010), particularly in caudal, lateral and intermediate subfields (Hyman et al., 1986; Mikkonen et al., 1999). Moreover, the initiation of tangles in the LEC has previously been theorized to be associated with interference between episodic associations (Hasselmo, 1994). Similarly, our data has relevance to our understanding of amnesia since amnesic patients with damage to the hippocampus and surrounding medial temporal lobe are unable to implicitly recognize target stimuli within familiarly patterned contexts unlike healthy adults (Chun and Phelps, 1999), an effect with similarities to those reported here in rats.
